# Retraction: Post grafting time significantly influences royal jelly yield and content of macro and trace elements

**DOI:** 10.1371/journal.pone.0275946

**Published:** 2022-10-21

**Authors:** The PLOS ONE Editors

The *PLOS ONE* Editors retract this article [1] because it was identified as one of a series of submissions for which we have concerns about authorship, competing interests, and peer review. We regret that the issues were not addressed prior to the article’s publication.

SNAK did not agree with the retraction. EKAT either did not respond directly or could not be reached.
